# Clinical proteomics and OMICS clues useful in translational medicine research

**DOI:** 10.1186/1477-5956-10-35

**Published:** 2012-05-29

**Authors:** Elena López, Luis Madero, Juan López-Pascual, Martin Latterich

**Affiliations:** 1 Centro de Investigación i + 12, Hospital 12 de Octubre, Av. De Córdoba s/n, 28040, Madrid, Spain; 2 Hospital Infantil Universitario Niño Jesús, Av. Menéndez Pelayo 65, 28009, Madrid, Spain; 3 Hospital Universitario 12 de Octubre, Av. De Córdoba s/n, 28040, Madrid, Spain; 4 Proteogenomics Research Institute for Systems Medicine, 11107 Roselle Street, San Diego, CA, 92121-1206, USA

## Abstract

Since the advent of the new proteomics era more than a decade ago, large-scale studies of protein profiling have been used to identify distinctive molecular signatures in a wide array of biological systems, spanning areas of basic biological research, clinical diagnostics, and biomarker discovery directed toward therapeutic applications. Recent advances in protein separation and identification techniques have significantly improved proteomic approaches, leading to enhancement of the depth and breadth of proteome coverage.

Proteomic signatures, specific for multiple diseases, including cancer and pre-invasive lesions, are emerging. This article combines, in a simple manner, relevant proteomic and OMICS clues used in the discovery and development of diagnostic and prognostic biomarkers that are applicable to all clinical fields, thus helping to improve applications of clinical proteomic strategies for translational medicine research.

## Introduction

### A. The post-genome era - advances in clinical proteomic research

Improved biomarkers are of vital importance for cancer detection, diagnosis and prognosis. While significant advances in understanding the molecular basis of disease are underway in genomics, proteomics will ultimately delineate the functional units of a cell: proteins and their intricate interactive networks and signalling pathways in health and disease.

Much progress has been made to characterize thousands of proteins qualitatively and quantitatively in complex biological systems by the use of multi-dimensional sample fractionation strategies, mass spectrometry (MS) and protein micro-arrays. Comparative/quantitative analysis of high-quality clinical biospecimens (e.g., tissue and biofluids) of the human cancer proteome landscape can potentially reveal protein/peptide biomarkers responsible for this disease by means of their altered levels of expression, post-translational modifications (PTMs), as well as different forms of protein variants. Despite technological advances in proteomics, major hurdles still exist at every step of the biomarker development pipeline [1-12].

In the post-genome era, the field of proteomics has incited great interest in the pursuit of protein/peptide biomarker discovery especially since MS has been shown to be capable of characterizing a large number of proteins and their PTMs

In complex biological systems, in some instances even quantitatively. Technological advances such as protein/antibody chips, depletion of multiple high abundance proteins by affinity columns, and affinity enrichment of targeted protein analytes as well as multidimensional chromatographic fractionation, have all expanded the dynamic range of detection for low abundance proteins by several orders of magnitude in serum or plasma, making it possible to detect the more abundant disease-relevant proteins in these complex biological matrices [13-21]. However, plasma and cell-extract based discovery research studies aimed at identifying low abundance proteins (e.g. some kinases) are extremely difficult. Therefore, it is necessary to develop significant technological improvements related to identifying this low abundance, although high biological impact molecules. Moreover, if these protein kinases to be studied contain PTMs, it is important to know that spatial and temporal factors can decrease the efficiency of our study (e.g. many kinases are regulated by phosphorylation of the activation loop, which then directly reflects cellular kinase activity).

Furthermore, proteomics has been widely applied in several areas of science, ranging from deciphering molecular pathogenesis of diseases, the characterization of novel drug targets, to the discovery of potential diagnostic and prognostic biomarkers, where technology is capable of identifying and quantifying proteins associated with a particular disease by means of their altered levels of expression [22-24] and/or PTMs [25-27] between the control and disease states (e.g., biomarker candidates). This type of comparative (semi-quantitative) analysis enables correlations to be drawn between the range of proteins, their variations and modifications produced by a cell, tissue and biofluids and the initiation, progression, therapeutic monitoring or remission of a disease state.

PTMs including phosphorylation, glycosylation, acetylation and oxidation, in particular, have been of great interest in this field as they have been demonstrated to being linked to disease pathology and are useful targets for therapeutics.

In addition to MS-based large-scale protein and peptide sequencing, other innovative approaches including self-assembling protein microarrays [28] and bead-based flow cytometry [29] to identify and quantify proteins and protein- protein interaction in a high throughput manner have furthered our understanding of the molecular mechanisms involved in diseases.

In summary, clinical proteomics has come a long way in the past decade in terms of technology/platform development and protein chemistry, to identify molecular signatures of diseases based on protein pathways and signalling cascades. Hence, there is great promise for disease diagnosis, prognosis, and prediction of therapeutic outcome on an individualized basis.

### B. Proteomic hindrances for discovery of true candidate biomarkers

Why is there such a disconnection between biomarker discovery using modern proteomic technologies and biomarker qualification requiring much more stringent analytical and clinical criteria? Several major obstacles have been suggested as being responsible for this discrepancy, including:

(a) technological variability within/across proteomic platforms;

(b) suitable/unsuitable biospecimen collection, handling, storage and processing;

(c) capacity/incapacity of credentialing biomarker candidates prior to costly and time-consuming clinical qualification studies using well-established methodologies;

(d) necessity for knowledge in the evaluation criteria required for these distinct processes in the pipeline and in regulatory science by the research community;

(e) insufficient publicly available high-quality reagents and data sets to the cancer research community;

(f) need for improved data analysis tools for the analysis, characterization, and comparison of large datasets and multi-dimensional data;

(g) necessity for proper experimental study design when performing studies involving clinical samples in biomarker studies.

**Figure 1:**
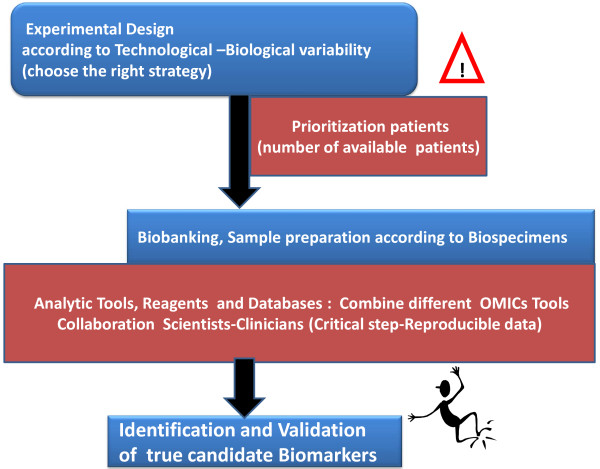
**Proteomic hindrances for discovery of true candidate biomarkers.** This figure illustrates, in a simple manner, relevant discovery aspects of true candidate biomarkers. Points to be considered are: **(a)** technological and biological variability within/across proteomic platforms; **(b)** suitable/unsuitable biospecimen collection, handling, storage and processing; **(c)** capacity/incapacity of credentialing biomarker candidates prior to costly and time-consuming clinical qualification studies using well-established methodologies; **(d)** the necessity for knowledge in the evaluation criteria required for these distinct processes in the pipeline and in regulatory science by the research community; **(e)** insufficient publicly available high-quality reagents and data sets to the cancer research community; **(f)** need for improved data analysis tools for the analysis, characterization, and comparison of large datasets and multi-dimensional data; and **(g)** necessity for proper experimental study design when performing studies involving clinical samples in biomarker studies. This implies a network-connectivity in relation to: **(h)** ensuring the choice of the correct strategy, **(i)** conclusion of the clinical proteomic research study when reaching a reprensative number of patients in order to achieve reliable data, **(j)** to always carry out inter- and intra-assays of your sample-preparations in order to reproduce your data, **(k)** to combine different OMIC-Tools to complement and verify the efficiency of your results, **(l)** Collaboration between clinicians and expert OMIC-scientists is necessary for succeess.

If proteomics is to be successfully introduced into clinical diagnostics, universally accepted metrics will be necessary at many steps along the way, to ensure that changes observed are attributable to biological states, not workflow variability. In addition, with the combination of different OMICS- technologies, more reliable data can be achieved. A high number of OMICS-combination-approaches are available for clinical research. It is always necessary to test different tools in order to raise a greater level of efficiency for your clinical study [30]. Figure 1 illustrates the proteomic hindrances for discovery of true (as opposed to surrogate) candidate biomarkers.

With regards to discovery, semi-quantitative proteomic methodologies routinely used for biomarker research between normal and diseased states are differential two-dimensional gel electrophoresis (2DGE), comparative label-free and labelling approaches [e.g., ^18^O labelling, Isotope Coded Affinity Tags, Isobaric Tag for Relative and Absolute Quantitation (iTRAQ), Stable Isotope Labelling with Amino Acids in Cell Culture (SILAC), Absolute Quantitation (AQUA), Multiple Reaction Monitoring (MRM)] followed by liquid chromatography mass spectrometry (LC-MS). Although such comparative analysis yields important information on possible changes as a result of disease, these current methods in clinical proteomics based, for the most part, on MS and its combination with 2DGE, chromatography or biobead technology, might have limitations related to the sensitivity concentration level.

#### Sample preparation

When using the previously mentioned proteomic tools, sample preparation is one of the most crucial processes in proteomic analysis and biomarker discovery in solubilized samples. Chromatographic or electrophoretic proteomic technologies are also available for separation of cellular protein components. There are, however, considerable limitations in currently available proteomic technologies as none of these allows for the analysis of the entire proteome in a simple step because of the large number of peptides, and because of the wide concentration dynamic range of the proteome in clinical blood samples. The results of any experiment undertaken depend on the condition of the starting material. Therefore, proper experimental design and pertinent sample preparation are essential for obtaining meaningful results, particularly in comparative clinical proteomics in which one is looking for minor differences between experimental (diseased) and control (non-diseased) samples [31].

Homogenization is one of the preparative steps employed for preparation of biological samples for proteomic analysis, and includes processes such as mixing, stirring, dispersing, or emulsifying in order to change the sample’s physical, but not chemical properties. Homogenization for proteomics incorporates five main categories: mechanical, ultrasonic, pressure, freeze-thaw, and osmotic/detergent lyses. Mechanical homogenization for tissues and cells can be accomplished by devices such as rotor–stator, and open blade mills (e.g., Warring blender and Polytron), or pressure cycling technology (PCT) such as French presses. Rotor– stator homogenizers can homogenize samples in volumes from 0.01 mL to l20 L depending on the tip and motor used.

For optimum results, the tissue should be cut into slices, the size of which is slightly smaller than the diameter of the applied stator, as larger samples may clog the generator’s inlet, making it impossible to achieve effective homogenization. Depending on the chemical resistance of a cutting tool, it is possible to homogenize samples under acidic or basic conditions in order to prevent degradation by endogenous enzymes. Heat transfer to the processed mixture is low to moderate and the process usually requires external cooling. Sample loss is minimal compared to PCT, where by means of a pressure-generating instrument (Pressure Bioscience, West Bridgewater, MA) alternating cycles of high and low pressure are applied to induce cell lysis [32,33].

In relation to protein solubilisation, proteins in biological samples are generally found in their native state associated with other proteins and often integrated as a part of large complexes, or into membranes. Once isolated, proteins in their native state are often insoluble. Breaking interactions involved in protein aggregation (e.g., disulfide hydrogen bonds, van der Waals forces, ionic and hydrophobic interactions) enables disruption of proteins into a solution of individual polypeptides, thereby promoting their solubilisation. However, because of the great heterogeneity of proteins and sample-source related interfering contaminants in biological extracts, simultaneous solubilisation of all proteins remains a challenge. Integration of proteins into membranes, and their association and complex formation with other proteins and/or nucleic acids hamper the process significantly. No single solubilisation approach is suitable for every purpose, and each sample and condition requires unique treatment. Sample solubilization can be improved by agitation or ultrasonification, but an increase in temperature should be avoided. The selection of the appropriate solubilisation protocol and buffers has specially been facilitated by the availability of commercial kits, although it is somewhat more expensive than routine reagent methods [34,35].

To avoid protein modifications, aggregation, or precipitation resulting in the occurrence of artifacts and subsequent protein loss, sample solubilization process requires the use, in the sample buffer of: (1) chaotropes (urea, thiourea, charged guanidine hydrochloride,-for e.g.-) that disrupt hydrogen bonds and hydrophilic interactions enabling proteins to unfold with ionizable groups exposed to solution; (2) ionic, non-ionic and zwitterionic detergents (SDS, CHAPS, or Triton X-100); (3) reducing agents that disrupt bonds between cysteine residues and thus promote the unfolding of proteins (DTT/dithioerythritol (DTT/DTE) or tributylphosphine (TBP) or tris-carboxy ethyl phosphine (TCEP)) and (4) protease inhibitors [36].

Although there is no general procedure to select an appropriate detergent, nonionic and zwitterionic detergents such as CHAPS and Triton X series are less denaturing than ionic detergents, and have been used to solubilise proteins for functional studies. On the other hand, ionic detergents are strong solubilizing agents that lead to protein denaturation. However, sodium cholate and deoxycholate are soft detergents compatible with native protein extraction, although variables like buffer composition, pH, salt concentration, temperature, and compatibility of the chosen detergent with the analytical MS procedure, and the way in which to remove it (by dialysis for example) are all crucial factors that need to be considered. Usually, tissue disruption and cell lyses require the combination of detergent and mechanical methodologies [35]. The proper use of the above reagents, together with the optimized cell disruption method, dissolution, and concentration techniques collectively determines the effectiveness of proteome solubilization methodologies.

All the previously detailed information, coupled to the use/study of blood, as a biospecimen in discovery research (a commonly used biospecimen which is highly complex and which has a wide dynamic range of protein concentrations), makes it is very difficult to discover (measure) low abundance proteins (potential biomarkers). One solution to this problem is to develop and apply nanotechnology in clinical proteomics, as well as the throughput of analytical measurement systems while lowering their cost. Not only does nanotechnology have the potential of fulfilling many criteria required for the advancement of clinical proteomics, essential changes in the physicochemical properties of substances on their conversion to the nanostructured state, but it has also made it possible to create efficient systems for drug delivery to targets.

In addition, blood cells offer unique insights into disease processes. Therefore, erythrocytes, granulocytes, monocytes, lymphocytes, and platelets are of special interest for clinical proteomics. Cytometry is currently widely used as an analytical tool for clinical cell analysis directly from anticoagulated whole blood and also for cell sorting to generate pure populations of cells from heterogeneous and highly integrated mixtures as are found in the majority of biological environments. Elispot, slide based cytometry, and tissue arrays together with high-content screening microscopy are further upcoming techniques in cytoproteomics. The major challenge for this type of preanalytical standardization is related to the use of fresh samples, either for direct multiparameter analysis of cellular proteomics in whole blood or body fluids without pre-separation, or for cell sorting and enrichment strategies for subsequent proteomic and functional genomic analysis [37].

### C. Nanotechnology to complement clinical proteomics

The identification of unique patterns of protein expression, or biomarkers, associated with a specific disease is one of the most promising areas of clinical proteomics. There is an urgent need to discover new biomarkers that are useful for early disease diagnosis. Recently, it has been recognized that the measurement of a panel of multiple biomarkers has the potential to achieve a much higher sensitivity and specificity compared with any single biomarker in the past. Moreover, the highest informative content is thought to reside in the low molecular weight (LMW), low abundance fraction of biological fluids. Nanotechnology offers new approaches to harvest low abundant panels of biomarkers. For cancers, if the disease can be detected prior to the onset of metastases, this can lead to a significant reduction in cancer deaths [38].

The envisioned role of nanotechnology is twofold:

(1) to provide access to previously inaccessible data as related to “-omic” technology components with unparalleled efficiency and resolution;

(2) to enable innovative therapeutic modalities that leverage the validated system biology outputs for exquisitely specific individualized therapy.

Systems biology has the potential for utilizing subtle biological clues (e.g. “-omic” technology components) for early detection of disease, predicting patient response to therapy, and identifying biomarkers to enable effective targeting of drug- delivery modalities to the disease site. The field of systems biology is still evolving, however there is strong evidence in scientific literature supporting the promise of nanotechnology as an enabling contributor for extracting the elusive “- omic” data for clinical analysis.

For example, investigators have recently shown the ability to reproducibly enhance the presence of the low molecular weight proteome from serum and plasma samples to differentiate the stages of disease as well as predict a patient’s response to therapy. As the utility of nanotechnology expands to other “-omic” technologies, the ability to compare and integrate multiple panels of data subsets will tremendously strengthen the validation process for biomarker identification. Furthermore, nanotechnology has already demonstrated a clinical impact upon drug-delivery strategies for a variety of ailments, particularly cancer indications.

The inherent scale of nanotechnology enables a library combining surface modifications (e.g. targeting moieties, charge modifications, stealth) of nanoparticulates, as well as control over size, shape, and other particle characteristics pending on particle material. This variety of options allows the rational design of personalized therapies that are predicated upon established biomarker evidence through system biology discovery, image analysis, mathematical modeling and access to effective chemotherapeutics and other agents [39]. The development of nanotechnology presents an unprecedented opportunity for point-of-care testing devices by enabling both greater analyt ical sensitivity and the ability to multiplex protein and nucleic acid marker evaluations in the same assay [40]. It is certain that nanotechnology has yet to impart an enabling contribution towards the overall movement to individualized medicine; thus, the potential of nanomedicine coupled to clinical proteomics remains undeniable.

Currently, one of the most promising nanotechnological proteomics under development for medical research is biosensor-based nanodiagnostics. An example of this is the development of a magneto-nano sensor protein chip and a multiplex magnetic sorter based on magnetic nanoparticles that allow rapid conversion of discrete biomolecule binding events into electrical signals, which can detect target molecules down to the single molecule level in less than an hour [41,42]. In consequence, nanotechnology in clinical proteomics today, implies a new medical research direction, dealing with the creation and application of nanodevices for carrying out proteomic analyses in the clinic. Nanotechnological progress in the field of atomic force microscopy facilitates clinical studies on the revelation, visualization and identification of protein disease markers, in particular of those with sensitivity of 10–^17^ M, much greater than the sensitivity of commonly adopted clinical methods. Also, at the same time, implementation of nanotechnological approaches into diagnostics permits the creation of new diagnostic systems based on the optical, electro-optical, electromechanical and electrochemical nanosensoric elements at high operating speed [42].

In summary, nanobiotechnology is a new focus in technological science. It plays a key role in the creation of nanodevices for the analysis of living systems on a molecular level. Moreover, nanomedicine allows for improved understanding of human life while using the knowledge on human organism at a molecular level. The use of nanotechnological approaches and nanomaterials opens new prospects for the creation of drugs and systems for their directed transport. Implementation of optico-biosensoric, atomic-force, nanowire and nanoporous approaches into genomics and proteomics will significantly enhance the sensitivity and accuracy of diagnostics and will shorten the time for diagnostic procedures, thus undoubtedly improving the efficiency of medical treatment.

### D. Bioinformatics : Useful for clinical proteomics

Computational biology covers a wide spectrum of techniques devoted to the generation and use of useful information from structure, sequence or relationships among biological analytes (DNA, RNA, proteins, macromolecular complexes, etc.). Those methods most useful in clinical studies, including biomarkers research, are chiefly the following:

–  Next Generation Sequencing (NGS) is recently being used in a detailed study of genes involved in ColoRectal Cancer (CRC).The authors demonstrated that sequencing of whole tumour exomes allowed prediction of the microsatellite status of CGC, and also, facilitating the putative discovery of relevant mutations. Additionally, NGS is applicable to formalin-fixed and paraffin embedded material, allowing the renewed study of relevant clinic material in the pathology departments [43,44].

– Once modified residues have been found in sequencing or proteomic studies, routine sequence-to-sequence and sequence-to-structure comparisons (MSA: multiple sequence analysis) allow to obtain valuable information about the functional implications related to the mutated residues in the protein context. Multiple alignments of proteins, and chiefly those based on the comparison of experimentally obtained three -dimensional atomic structures (structural alignments), are a very valuable source of information related to the evolutionary strategies. This is then followed by the different members of a family of proteins to conserve or modify their functions and structures [45].

The analysis of structural alignments allows the detection of at least three types of regions or multiple alignment positions according to conservation: (a). *Conserved positions*, usually the key for function or structure maintenance. (b). *Tree- determinant residues*, conserved only in protein subfamilies and related to family- specific active sites, substrate binding sites or protein-protein interaction surfaces. These sites contain essential information for the design of family-specific activator or inhibitor drugs [46]. And (c), positions that correspond to compensatory mutations that stabilize the mutations in one protein with changes in the other (*Correlated mutations*). These sites are very effective for the detection of protein- protein interaction contacts [47]. These last ones allow for the selection of the correct structural arrangement of two proteins based on the accumulation of signals in the proximity of interacting surfaces.

– Because of the sequence-to-structure comparison, and in absence of experimental crystal structures, the homology modelling methods, (also called comparative modelling or knowledge-based modelling), can develop a 3D model from a protein sequence based on the structures of a crystallized homologous protein. The method can only be applied to proteins with a common evolutionary origin: as only for proteins that are hypothesized to be homologous, this assertion implies that their three-dimensional structures are conserved to a greater extent than their primary structures. In the event where good homology hypothesis cannot be seconded, alternative methods can be applied in order to obtain a putative 3D structure. These procedures, known as “far-homology modelling” or “threading” methods, provide structures with lesser confidence compared to those generated using homology modelling methods.

– Data on the 3D structure of the active centre of a protein of interest and/or its natural ligands can be used as a basis for the design of effective drugs. This rational drug design is usually performed via multiple docking experiments in the active centre of the protein of interest. This requires the use of advanced software such as Autodock-4 [48]. Algorithms such as Autodock-4 allow the evaluation of not only the docking to a rigid model of the active centre, and also a certain mobility and adaptation of the side chain of enzyme residues to the ligand shape. Commonly, all the calculated binding conformations to the target protein obtained in every docking run are clustered according to scoring criteria (as “the lowest binding energy model” or “the lowest energy model representative of the most-populated cluster”) and sorted according to their estimated free energy of binding. These computer strategies are a useful cost-reducing tool to prospect and model new molecules with potential inhibiting properties or even successful future drugs. Lately, the rational drug design approach has been used for putative cancer therapies, in particular the pharmacological reactivation of mutant p53 [49]. This promising strategy implies the simultaneous use of several ways for the identification of small molecules that target mutant p53, including “*de novo*” design and screening of chemical libraries.

– To conclude this section, molecular dynamics (MD) techniques are routinely used to obtain refined models for protein structure, protein-protein and protein- ligand interactions. MD is a computational simulation technique in which atoms within molecules are allowed to interact for a period of time according to the principles of physics. In the case of proteins, the relevant forces taken into account are the electrostatic interactions (of attraction or repulsion), Van der Waals interactions, and the properties of the covalent bond (length, angle, and dihedral angle). As a rule, simulation times for macromolecular protein complexes are up to 20 ns and the number of atoms of the simulated systems is in the order of up to 250,000, including solvent molecules. MD tools have been used to simulate the individual behaviour of small protein or peptides [50], protein-protein interfaces and ligand-protein relationship in catalytic macromolecular complexes with GTPase activity [51,52] or kinases involved in cell signalling pathways (e.g. Src tyrosine kinase [53] or the protein kinase B/Akt [54]).

### E. Sample biobanking omplementation necessary for clinical proteomics

A Biobank contains several hundred thousand samples from a broad range of anatomic sites, diseases and with diverse ethnic representation. All biospecimens are obtained using stringent standard operating procedures and ethical protocols to provide assurance to the researcher that the materials will meet their scientific needs.

In order to require tissue samples with accompanying clinical outcome data, it is necessary to maintain a BioReserve repository of frozen and fixed tissues with patient follow-up data.

Through Biobank and BioReserve repositories, it is, thus, possible to provide a rapid delivery of human tissue, biofluids and tissue derivatives that best meet the research requirements. Moreover, during a standard collection protocol for sample biobanking, human tissues or bodyfluids and clinical data can also be custom collected to meet unique requirements. Each donor site uses a standardized clinical data form and pathologic data is classified using codes for anatomic site, morphology and behaviour [55].

As a final check, clinical data management associates review records for each case to ensure complete and consistent data. The stringent evaluation and classification process ensures that scientists receive clinically relevant data to help them in their research. Each biospecimen is assessed using uniform quality assurance tests. The pathologists independently confirm the anatomic site and diagnosis for each tissue procured. In addition, the lab researchers assess the RNA, DNA, proteins etc. integrity of each tissue received. This information is pr ovided to scientists before purchase so they can accurately select the samples which will best meet their needs. This reduces the number of failed OMICS and clinical experiments due to inappropriate or poor quality samples [56].

## Considerations and future needs

### A. The conventional biomarker development pipeline

It is necessary to integrate genomics, proteomics, nanobiotechnology/nanomedicine, bioinformatics and biobanking-sample methodologies with clinicians. The mapping of the human genome represents a real milestone in medicine and has led to an explosion in discoveries and translative research in life sciences. Indeed, this important knowledge base has enabled rapid development in the areas of diagnostics, gene therapy, new drug targets discovery, and personalized therapies [57,58]. The expansion of biological knowledge through the Human Genome Project (HGP) has also been accompanied by the de velopment of new high throughput techniques, providing extensive capabilities for the analysis of a large number of genes or the whole genome. The completion of the human genome, however, has presented a new and even more challenging task for scientists: the characterization of the human proteome. Unlike the genome project, there are major challenges in defining a comprehensive Human Proteome Project (HPP) due to (a) a potentially very large number of proteins with PTMs, mutations, splice variants, etc.; (b) the diversity of technology platforms involved; (c) the variety of overlapping biological “units” into which the proteome might be divided for organized conquest; and (d) sensitivity limitations in detecting proteins present in low abundances.

The conventional biomarker development pipeline involves a discovery stage followed by a qualification stage (commonly known as biomarker validation) on large cohorts, prior to clinical implementation and designing complementary OMICs strategies. In common practice, the discovery stage is performed on a MS-based platform for global unbiased sampling of the proteome, while biomarker qualification and clinical implementation generally involve the development of an antibody-based protocol, such as the commonly used enzyme linked ELISA assays. Although this process is potentially capable of delivering clinically important biomarkers, it is not the most efficient process as the latter is low-throughput, very costly and time-consuming. In many cases, affinity reagents for novel protein candidates do not even exist and it is difficult to multiplex targets without creating significant interferences and cross-reactivity. These limitations of immunoassays have called for the development of alternative approaches. The recent surge in the advance of proteomic technologies centering on targeted MS and protein microarrays has provided great opportunities for researchers to use them as “bridging technologies” for clinical proteomic and OMICS investigation of disease-relevant changes in tissues and biofluids.

Some recent studies that combine rigorous study design with a focused mass spectrometry approach, promise to streamline the discovery and validation process [59,60]. These studies deviate from the traditional brute-force discovery efforts, geared to find minute differences between often complex samples, to employ pre-selection and MRM-based quantification strategies. This approach significantly enhances the fidelity of detecting significant differences between even low abundance biomarkers. To put it into the perspective of the proverbial “needle in a haystack” analogy the “haystack” has not become smaller; however, the pre-selection of potential biomarkers of significance has provided the research community with a “magnet” to make the quest for finding the needle more efficient.

On the other hand, apart from restructuring the biomarker development pipeline, it will now become critical to introduce regulatory science to the proteomics together with nanotechnology/nanobiomedicine and bioinformatic research (OMICS technologies in general) with clinical chemistry community so that all these technologies can be translated from the laboratory to the clinic.

### B. The relevant role of clinical laboratories

Clinical laboratories have an important role, and clinical scientists undoubtedly play an important part in the analytical validation of diagnostic tests and are thus required to routinely verify (confirm) previously cleared/approved tests by the regulatory agency in their facilities. Post-market analytical validation is routinely performed by clinical researchers via evaluation tests (strategies, instruments, positive and negative controls, reagents, etc.), which complies with regulations, specifications, or conditions. These tests typically involve precision, accuracy, linearity and lower limits of detection and quantification.

On setting up a method for an approved multiplex protein assay using a patient -specific “score”, clinical scientists should consider the way in which to perform studies to validate the score. One approach may involve running an adequate number of positive and negative patients to assess the performance of such a “score” in their diagnosis in comparison with their medical charts and final clinical diagnosis. Additionally, international collaboration provides an effective means by which to educate key clinical laboratory audiences about the need for and use of common technologies and standards in proteomic and OMICS workflows and to share knowledge and experience on commonly interesting targets, assays and new technologies.

On the other hand, the reduction universally observed in test development and research activities represents, in part, a shift from laboratories making their own reagents and immunoassays to the purchase of the majority of them from an in vitro diagnostics company.

This is not an entirely negative development. External quality assessment and proficiency testing data clearly demonstrate the benefits of automation, including much improved precision, and there are benefits of scale in centralizing test development processes. Nevertheless, and as we previously mentioned, clinical laboratories should play an active role in the final evaluation of assays and in the study of their clinical utility in relation to their patients.

When considering requirements for the successful introduction of new diagnostic tests, it is helpful to review the general criteria that must be met (see Table 1), focusing on the roles of both research and specialist laboratories and the somewhat different requirements of high- throughput routine laboratories [61-64].

**Table 1:** Tips for the discovery of true candidate biomarkers at clinical laboratories

**Necessity**	**Suggestion**
Clinically clearly understood	Direct comparison with the existing best practice in the population for which it is intended
Well-characterized clinical specimens for discovery the relevant clinical population	Several factors have to be taken into account when collecting specimens for the studies of new biomarkers, whether for a specific clinical study or for a biobank in order to enable interpretation of results and ensure appropriate matching of patient and health controls
Well-validated discovery platform which is robust and reliable	The use of internal standards for identifying specific components and quality control via proteomic –mass spectrometry and OMICS strategies is critical.
Clinical evidence for the true candidate biomarker	Take into account: (a) which is the association of our candidate-biomarker with the relevant disease, (b) which is the assessment of clinical utility and impact, (c) which are the circumstances where use of the test would be unjustified and (d) Make a rigorous early investigation of the specific pre-analytical factors which might influence interpretation of the resulting data

This table illustrates the necessities for the successful transition when discovering true biomarkers from the research environment (lab) to the clinical applications and utilities.

### C. Integration of OMIC-scientists experts with clinicians

The ultimate goal for translational medicine is its capacity to perform assays in various clinical samples at multiple levels: DNA (genome), RNA (transcriptome) and protein (proteome) coupled to bioinformatics and nanotechnology/nanobiomedicine and others, using

the knowledge and technologies resulting from large-scale projects. This workflow provides a genetic basis and a good opportunity for the community to characterize and quantify proteins (reflecting genetic alterations if detectable) and their alterations and PTMs in the cell.

It is critical to define the final purpose of a biomarker or biomarker pattern at the onset of the study and to select the case and control samples accordingly. This is followed by the experiment design, starting with the sampling strategy, sample collection, storage and separation protocols, choice and validation of the quantitative profiling platform followed by data processing, statistical analysis and validation workflows. Biomarker candidates arising after statistical validation should be submitted for further validation and, ideally, be connected to the disease mechanism after their identification. Since most discovery studies work with a relatively small number of samples, it is necessary to assess the specificity and sensitivity of a given biomarker-based assay in a larger set of independent samples, preferably analyzed at another clinical centre. Targeted analytical methods of higher throughput than the original discovery method are needed at this point and LC-tandem mass spectrometry is gaining acceptance in this field [65,66].

The resulting proteomic evidence will corroborate or complement the genetic aberrations detected in samples, such as tumours, providing deeper understanding of cancer and other diseases in the context of biological and clinical utility. The integration and interrogation of the proteomic and genomic data (and OMICS data in general) will provide potential biomarker candidates, which will be prioritized for downstream targeted proteomic analysis. These biomarker targets will be used to create multiplex, quantitative assays for verification and pre-screening to test the relevance of the targets in clinically relevant and unbiased samples. The outcomes from this approach will provide the community with verified biomarkers which could be used for clinical qualification studies; high quality and publicly accessible datasets; and analytically validated, multiplex, quantitative protein/peptide assays and their associated high quality reagents for the research and clinical community.

It is also important to state that in order to develop clinical proteomic and OMICS applications using the identified proteins (with and without PTMs), collaboration between research scientists, clinicians, diagnostic companies and industry, and proteomic experts is essential, particularly in the early phases of the biomarker development projects. Also, complementing the data with other OMICS tools is crucial. The proteomics modalities currently available have the potential to lead to the development of clinical applications, and the channelling of the wealth of the information produced towards concrete and specific clinical purposes is urgent [65-67].

New biomarkers can be taken from research by experts in OMICS and clinicians into routine practice, provided there is sound evidence of clinical utility, funding can be assured, mechanisms are in place to ensure that the test is done only for those likely to benefit, analytical procedures are simple and robust, and quality is verified through internal quality control and efficiency testing procedures. For these requirements to be met in a timely manner for a specific biomarker, it is essential to learn from past mistakes and perhaps to think differently in the future.

For the future, greatly improved involvement and collaboration from all interested parties – including experts in discovery and assay development, in health policy, in clinical trial units, in the diagnostics industry and in laboratories responsible for providing clinical testing – will almost certainly lead to earlier identification and implementation of promising new biomarkers [68-71].

## Summary of important clues when applying clinical OMICS strategies for translational medicine research

(I) Standardizing sample preparation procedures for each sample (e.g. blood, plasma/serum, etc.), is critical for obtaining reliable biomarkers and building a biomarker pattern, since slight changes in a given sample preparation could lead to very different protein profiles.

(II) Clinical Proteomics and Bioinformatics for Translational Medicine research studies include steps for improvements that should be made and well-controlled in: (a) analytical tools and biobanking-samples, (b) discovery, (c) validation, (d) clinical application, and (e) post-clinical application appraisal. It is likely that most, if not all, of the components that are necessary for clinical success are either readily available, or could be allocated with more rigorous research standards and efforts supported by our scientific community, clinicians, health agencies including hospitals, diagnostic companies, and industry. Enthusiasm for the clinical impact of proteomics may need to be tempered, at present, until robust evidence can be obtained, but some clinical successes will eventually be feasible.

(III) The rapid proliferation of Nanotechnology/nanobiomedicine and the implementation of sample-Biobanking are revolutionizing science and technology. There is marked interest regarding the use of nanotechnologies in medicine coupled to clinical proteomics, and to complement OMICS tools in general. Therefore clear advances are appearing for the discovery of true candidate biomarkers.

(IV) However, and as a general rule, it must be taken into account as a very important conclusion, that without: (a) the correct study design, (b) the correct and complementary strategies (c) implementation of robust analytical methodologies and (d) the necessity for collaboration among expert OMICS scientists together with clinicians and the industry, the efforts, efficiency and expectations to make true candidate biomarkers a useful reality in the near future can easily be hindered.

## Abbreviations

AQUA: Absolute quantitation; CID: Collision-induced dissociation; CRC: Colorectal cancer; Da: Dalton (molecular mass); DIGE 2-D: Fluorescence difference gel electrophoresis; 2DGE: two-dimensional gel electrophoresis; ECD: Electron capture dissociation; ESI: Electron spray ionization; ETD: Electron transfer dissociation; FT-ICR: Fourier transform-Ion cyclotron resonance; HILIC: Hydrophilic interaction chromatography; HGP: Human genome project; HPP: Human proteome project; HPLC: High-performance liquid chromatography or high-pressure liquid chromatography; H_3_PO_4_: Phosphoric acid; ICR: Ion cyclotron resonance; iTRAQ: Isobaric tag for relative and absolute quantitation; IMAC: Immobilized metal affinity capture; IT: Ion trap; kDa: Kilodalton (molecular mass); LC: Liquid chromatography; LC-MS: Liquid chromatography mass spectrometry; LMW: Low molecular weight; MALDI: Matrix-assisted laser desorption/Ionization; MD: Molecular dynamics; MOAC: Metal oxide affinity chromatography; Mr: Relative molecular mass (dimensionless); MRM: Multiple reaction monitoring; MS: Mass Spectrometry; MS/MS: Tandem mass spectrometry; MSA: Multiple sequence analysis; m/z: Mass to charge ratio; NGS: Next generation sequencing; PCT: Pressure cycling technology; PTM: Post-translational modification; SILAC: Stable isotope labelling with amino acid in cell culture; SIMAC: Sequential elution from IMAC; TiO_2_: Titanium dioxide; TOF: Time of flight; ZrO_2_: Zirconium dioxide; 3D: Structure three-dimensional structures; O labelling: Quantitative label-free and labelling approach.

## Competing interests

The authors declare that they have no competing interests.

## Authors’ contributions

Authors EL, LM, JLP and ML carried out Clinical Proteomics and Translational Medicine studies for this short-review, in order to develop future Oncohematology Proteomic-OMICS research studies and publish this article. All authors read and approved the final manuscript.
